# Environmental correlates of internal coloration in frogs vary throughout space and lineages

**DOI:** 10.1002/ece3.3438

**Published:** 2017-10-03

**Authors:** Lilian Franco‐Belussi, Diogo Borges Provete, Classius De Oliveira

**Affiliations:** ^1^ Department of Biology São Paulo State University (UNESP) São José do Rio Preto São Paulo Brazil; ^2^ FAPESP postdoctoral fellow Department of Environmental Sciences Federal University of São Carlos Sorocaba São Paulo Brazil; ^3^ Gothenburg Global Biodiversity Centre Göteborg Sweden; ^4^ Present address: Graduate program in Biotechnology and Environmental monitoring CCTS Federal University of São Carlos Sorocaba São Paulo Brazil; ^5^ Present address: Instituto de Biociências Universidade Federal de Mato Grosso do Sul Campo Grande Mato Grosso do Sul Brazil

**Keywords:** anurans, internal melanin, photoperiod, temperature, UV‐B

## Abstract

Internal organs of ectotherms have melanin‐containing cells that confer different degrees of coloration to them. Previous experimental studies analyzed their developmental origin, role in immunity, and hormonal regulation. For example, melanin increases with ultraviolet radiation (UV) and temperature in frogs and fish. However, little is known about how environmental variables influence the amount of coloration on organs among amphibian species over a large spatial extent. Here, we tested how climatic variables (temperature, UV, and photoperiod) influence the coloration of internal organs of anurans. We recorded the level of melanin pigmentation using four categories on 12 internal organs and structures of 388 specimens from 43 species belonging to six anuran families. Then, we tested which climatic variables had the highest covariation with the pigmentation on each organ after controlling for spatial autocorrelation in climatic variables and phylogenetic signal in organ coloration using the extended version of the RLQ ordination. Coloration in all organs was correlated with the phylogeny. However, the coloration of different organs was affected by different variables. Specifically, the coloration of the heart, kidneys, and rectum of hylids, *Rhinella schneideri*, some *Leptodactylus*, and *Proceratophrys* strongly covaried with temperature and photoperiod, whereas that of the testicle, lumbar parietal peritoneum, lungs, and mesenterium of Leiuperinae, Hylodidae, *Adenomera*, and most *Leptodactylus* had highest covariation with UV‐B and temperature. Our results support the notion that melanin pigmentation on the surface of organs of amphibians has an adaptive function conferred by the protective functions of the pigment. But most importantly, internal melanin seems to respond differently to climatic variables depending on the lineage and locality in which species occur.

## 1 INTRODUCTION

Vertebrates have a wide variety of body color patterns whose evolution is shaped by both natural and sexual selection (Cuthill et al., 2017). In addition, the internal organs and structures of ectotherms can also have different color patterns, which are given by different pigments. One of these pigments is melanin, which occurs in cells called melanocytes. These cells produce and store melanin (Agius & Roberts, 2003; Oliveira & Franco‐Belussi, 2012) and are similar to skin melanocytes (Franco‐Belussi, Castrucci, & Oliveira, 2013; Zuasti, Jiménez‐Cervantes, García‐Borrón, & Ferrer, 1998). Melanocytes occur in internal organs and membranes of amphibians (Oliveira & Franco‐Belussi, 2012) and fish (Agius, 1981). The functions of melanin pigmentation on the surface of organs are not yet well understood (see Oliveira & Franco‐Belussi, 2012). However, melanin is known to be involved in protection against bacteria and free radicals in ectotherms (Whitten & Coates, 2017; Wolke, George, & Blazer, 1985). Accordingly, previous experimental studies have established a link between the amount and distribution of melanin pigmentation on organs to temperature variation (Santos, Franco‐Belussi, Zieri, Borges, & Oliveira, 2014), ultraviolet B (UV‐B) radiation (Franco‐Belussi, Sköld, & Oliveira, 2016), bacterial infections (Franco‐Belussi et al., 2013), and the joint effect of UV‐B and bacteria (L. Franco‐Belussi et al. *in prep*.).

Environmental variables influence physiological and behavioral aspects of ectotherms (Hillman, 2009). Temperature can alter the internal coloration in anurans, due to the capacity of melanin to absorb visible and infrared radiation and transform them into heat (Cesarini, 1996). For example, the metabolism of hibernating amphibians at low temperatures is reduced, increasing melanin in liver cells as a consequence (Barni et al., 1999). The factors that increase liver pigmentation during long‐term hibernation include hypertrophy, hyperplasy, and melanosome accumulation in melanomacrophages to protect the organ against free radicals, as a way to compensate for the reduced activity of hepatocytes, whose function changes to energy storage (glycogen and lipids) during the winter (Barni et al., 2002). Conversely, high temperature decreases liver pigmentation in anurans in the short term (Santos et al., 2014), making cells less prone to absorb heat. Thus, species inhabiting warm localities may possibly have less pigmentation as an adaptive response to environmental temperature (see Santos et al., 2014). Additionally, daily thermal fluctuation decreased the growth rate of three larval anuran species, but the metabolic rate differed among them (Kern, Cramp, & Franklin, 2015). Consequently, it may be possible that internal pigmentation also varies among species as a result of the change in metabolic rates due to temperature variation, but this hypothesis remains to be tested.

Ultraviolet (UV) radiation is one of the factors responsible for worldwide amphibian declines (Blaustein, Kiesecker, Chivers, & Anthony, 1997). Exposure to UV affects embryonic development by promoting DNA damage, which may cause morphological changes and tadpole mortality, resulting in population declines (Lipinski, Santos, & Schuch, 2016), and also affects the immune system of adults (Ceccato, Cramp, Seebacher, & Franklin, 2016). Previous studies have also found that anuran species respond differently to UV (reviewed in Blaustein & Belden, 2003). For example, species that deposit eggs directly in open, shallow aquatic habitats are particularly more sensitive to the deleterious effects of UV (Blaustein & Belden, 2003) than those that lay eggs elsewhere, such as in foam nests in burrows (reviewed in Blaustein et al., 1998). Accordingly, amphibians have several defense mechanisms against UV exposition that include behavioral avoidance and both physiological and molecular mechanisms (Blaustein & Belden, 2003; Schuch, Lipinski et al., 2015). Internal melanin also seems to protect internal organs against the genotoxic effects of UV, because it is usually deposited around the cell nucleus in order to protect the DNA by absorbing the radiation and transforming it into heat (Roulin, 2014). Internal organ's coloration can also respond to UV radiation. For example, short‐term (e.g., 24 hr) exposure to UV‐B increases the coloration in both skin and organ's surface in adult anurans (Franco‐Belussi et al., 2016). Importantly, the consequences of exposure to UV are long lasting and not only affect amphibians during daylight, as UV induces DNA damage resulting in the production of cyclobutane pyrimidine dimers (CPD), mutation, and cell death in the long term (Blaustein & Belden, 2003; Schuch, Dos Santos et al., 2015). However, there are no data to show how much of UV radiation actually penetrates the skin. Nonetheless, no study has tested the effects of UV on the coloration of internal organs of multiple species (but see Franco‐Belussi et al., 2016 for *Physalaemus nattereri*), which could also potentially play a role in determining amphibian declines.

Another environmental variable that alters the skin coloration of fish and anurans is photoperiod. Light disperses melanin granules in cells of amphibians, increasing coloration (Moriya et al., 1996). Previous experiments showed that light intensity and time of exposure promote darkening of fish skin (Han, Xie, Lei, Zhu, & Yang, 2005). Also, the secretion of melanin‐concentrating hormone (MCH) increases in fish after long photoperiods (Lyon & Baker, 1993). The MCH is involved in the aggregation of melanin granules (Fujii, 2000), which makes the animal coloration lighter. However, little is known about how the variation in photoperiod throughout geographical space influences the coloration of visceral organs in anurans.

The effects of environmental factors on internal coloration have been thoroughly analyzed under experimental conditions in a few species (e.g., Franco‐Belussi et al., 2016; Santos et al., 2014). The main goal of these studies was to demonstrate the mechanistic basis of the responses of pigmentary cells to climatic factors. Additionally, a large‐scale study using 32 Neotropical anuran species found that closely related species have similar degrees of coloration on the heart, testes, lumbar parietal peritoneum, and lumbar nerve plexus (Provete et al., 2012). These authors also found that some families, such as Hylidae, Leptodactylidae, and Hylodidae, have more variation in the amount of coloration. Similarly, the amount of pigmentation also varied among organs, whereas the pigmentation on the pericardium, mesenterium, vertebral column, rectum, lungs, cardiac blood vessels, kidneys, and renal veins was more labile than in the heart, testes, lumbar parietal peritoneum, and lumbar nerve plexus. These results suggested that the role of melanin in each organ may vary, but did not point to its underlying causes. Here, we take advantage of these results and expand them to ask whether climatic factors, which experiments previously found to affect the amount of melanin on organs, may covary with differential coloration levels on organs over a large spatial extent. While proving the mechanistic link between climatic variables and the response of pigment cells, laboratory experiments are prohibitive to perform on large numbers of species. Conversely, correlational studies can be carried out with dozens of species and allow detecting patterns in large datasets, but cannot isolate specific mechanisms. Furthermore, the results of correlational studies can guide future experimental studies.

Here, we build upon our previous study (Provete et al., 2012) to test which climatic variables (temperature, UV, and photoperiod) have the highest covariation with coloration on the surface of internal organs in anurans using a three‐table ordination method called RLQ (R‐mode Linked to Q‐mode; Dolédec, Chessel, ter Braak, & Champely, 1996; Pavoine, Vela, Gachet, Belair, & Bonsall, 2011). Internal melanin can possibly confer adaptive advantages for species in response to environmental variables. Therefore, we hypothesize that coloration will respond similarly to climatic variables, regardless of the organ, as pigment cells belong to the same lineage (melanocytes). We also expect that groups of species occurring in the same region will respond differently to climatic variables, depending on their life history characteristics. For example, diurnal species tend to have a great amount of internal melanin, because during this period the UV incidence and temperature are higher.

## 2 MATERIALS AND METHODS

### 2.1 Specimen sampling

Anuran species were collected by hand when calling and using pitfall traps near breeding sites in 21 localities in the states of São Paulo and Goiás from October to March between 2006 and 2015. Specimens were housed at the collection of the Laboratório de Anatomia—UNESP. At least five adult males of each species were used for the analysis of pigmentation. The specimens were anesthetized with 5 g/L of benzocaine and dissected to expose the organs. All procedures followed the recommendations of the Brazilian College of Animal Experimentation (COBEA) and the Ethics Committee of our university (Protocol #70/07 CEEA).

**Figure 1:**
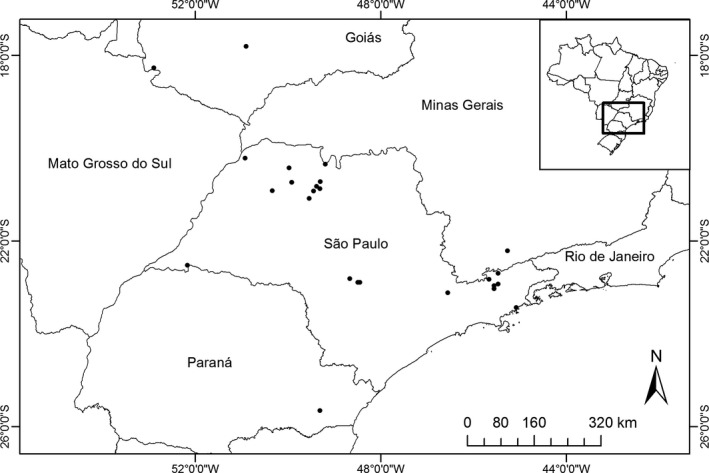
Map showing the sites sampled in this study and those to which we obtained trait data from the literature or museum specimens

We also analyzed additional specimens collected by hand and using pitfall traps from five localities in the states of Paraná, São Paulo, and Minas Gerais, which were deposited in the amphibian collections of the Departamento de Zoologia e Botânica, UNESP (DZSJRP) and Coleção Científica do Laboratório de Zoologia, Universidade de Taubaté (CCLZU; Figure 1). In total, we had 388 specimens from 43 species belonging to six families. Species had different sample sizes because same of them (e.g., *Dendropsophus minutus*) were widely distributed throughout our sampling sites, while others were restricted to a few localities (e.g., *Hylodes*). Thus, we could also investigate whether there is a spatial pattern in the effect of climatic variables on organ coloration within species.

### 2.2 Morphological data

**Figure 2:**
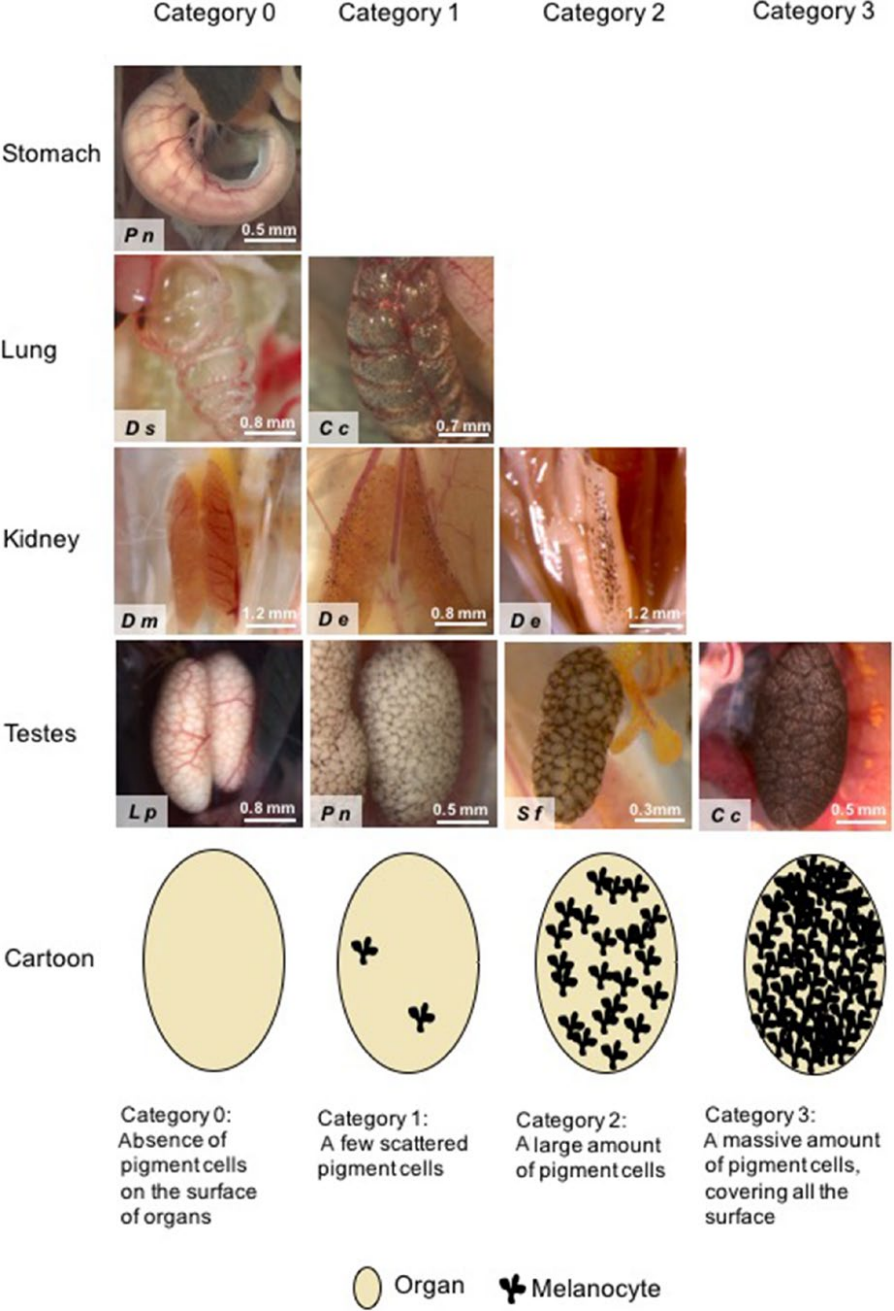
Coloration categories in a sample of organs, showing the naturally observed variation in coloration in the same organ among species. Some organs (e.g., stomach) do not have pigmentation in any species, while other (e.g., lung) have a narrow, medium (e.g., kidneys), and wide variation (e.g., testes) in coloration among species. Cc, *Crossodactylus caramaschii*; De, *Dendropsophus elianeae*; Dm, *Dendropsophus minutus*; Dn, *Dendropsophus nanus*; Ds, *Dendropsophus sanborni*; Lp, *Leptodactylus podicipinus;* Pn, *Physalaemus nattereri;* Sf, *Scinax fuscomarginatus*. Cartoon showing the scheme used to categorize coloration on the surface of internal organs, based on Franco‐Belussi et al. (2009)

We recorded the distribution of visceral melanocytes on the surface of 12 organs and structures using a Leica stereoscopic microscope (MZ16), coupled with an image capture system, namely: heart, lungs, rectum, lumbar parietal peritoneum, kidneys, testes, cardiac blood vessels, pericardium, renal veins, vertebral column, lumbar nerve plexus, and intestinal mesenterium. Those were the organs that had some degree of pigmentation and exhibited phylogenetic signal in a previous study (Provete et al., 2012). For each specimen, we recorded the intensity of coloration on these organs/structures (see full dataset in Franco‐Belussi, Provete, & Oliveira, 2017), following the protocol of Franco‐Belussi, Zieri, Santos, Moresco, and Oliveira (2009). Briefly, the intensity of organ coloration was divided into four categories, ranging from absence to entirely colored, as follows: Category (0) absence of pigment cells on the surface of organs, in which the usual color of the organ is evident; Category (1) a few scattered pigment cells, giving the organ a faint coloration; Category (2) a large amount of pigment cells; and Category (3) a massive amount of pigment cells, rendering an intense coloration to the structure, changing its usual color and superficial vascularization (Franco‐Belussi et al., 2009). Thus, for a given organ or structure, each individual could display four categories of coloration: 0, 1, 2, or 3 (Figure 2). This scheme of categorization is analogous to one used to describe the coloration in anuran testes by Grant et al. (2006). Coloration category was assessed in a double‐blind fashion. Pictures of some organs and regions are freely available in Morphobank at https://doi.org/10.7934/p701.

### 2.3 Climatic data

We extracted seven bioclimatic variables related to temperature from WorldClim v. 1.4 (Hijmans, Cameron, Parra, Jones, & Jarvis, 2005) for the 26 localities: BIO2 (Mean Diurnal Range), BIO3 (Isothermality), BIO4 (Temperature Seasonality), BIO5 (Max Temperature of Warmest Month), BIO6 (Min Temperature of Coldest Month), BIO7 (Temperature Annual Range), and BIO10 (Mean Temperature of Warmest Quarter). Data for photoperiod for each locality consist of a grand mean of monthly average of minutes of light hours between November 2012 and February 2013. This period is when most specimens were collected (the rainy season). Data were obtained from the Brazilian National Observatory (BRASIL, 2016). Data for UV‐B radiation were extracted from the glUV database (Beckmann et al., 2014). This database was generated using daily measurements of remotely sensed UV‐B levels from the Aura‐OMI satellite mission between 2004 and 2013, with a spatial resolution of 15 arc minutes. Specifically, we extracted the sum of monthly mean UV‐B during the highest quarter (UV‐B5), which is the period of the year in which frogs are most active and subjected to the effects of UV‐B.

### 2.4 Species phylogeny

**Figure 3:**
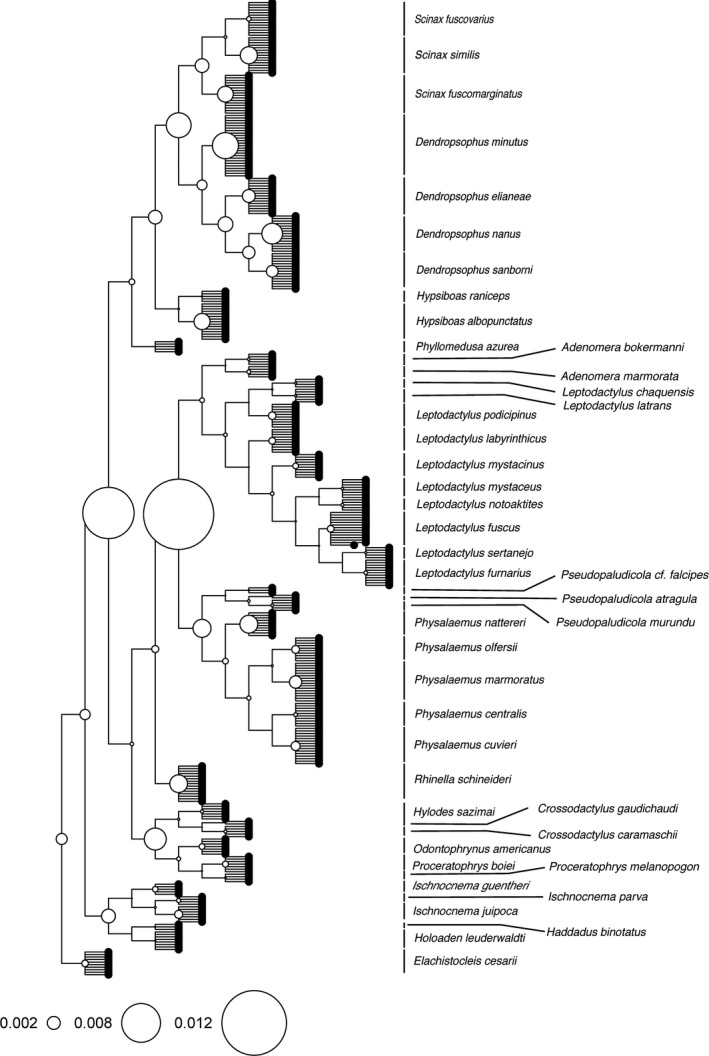
Phylogeny of 43 species from six families showing the result of the decomposition of the diversity of organ coloration along the nodes of the phylogeny considering all organs together. The size of circles represents the quadratic entropy. The diversity (measured as Rao's quadratic entropy) is skewed toward the root of the phylogeny, indicating phylogenetic signal. The topology is based on Pyron (2014), and individuals of each species (388 total) were included as polytomies at the respective species level

The phylogeny for the species to which we had trait data was pruned from the dated phylogeny of Pyron (2014) for amphibians. This phylogeny was inferred based on nine nuclear and three mitochondrial genes for 3,309 species, with average 20% of completeness. To this topology, we added each individual as a polytomy to its corresponding species with branch length equal to unit (Figure 3). As our trait is ordinal, we could not simply calculate its standard error to account for intraspecific variation. Also, as the method that decomposes the trait diversity along the nodes of the phylogeny (Pavoine, Baguette, & Bonsall, 2010) only considered tree topology, this is an adequate way to incorporate information at the individual level to our analysis.

### 2.5 Statistical analyses

#### 2.5.1 Data handling and exploratory data analysis

We standardized all environmental variables to zero mean and unit standard deviation prior to analysis. Posteriorly, we tested for multicollinearity (Zuur, Ieno, & Elphick, 2010) and removed environmental variables with variation inflation factor (VIF) higher than 10. The reduced variables were BIO2, BIO7, BIO10, UV‐B, and photoperiod (see Franco‐Belussi et al., 2017). We then tested for spatial autocorrelation (SAC) in the environmental variables (Legendre & Legendre, 2012). All environmental variables were significantly spatially autocorrelated, with global Moran's *I* varying between 0.154 and 0.485. This result suggested that the environmental variables were spatially structured, so nearby sites had more similar environment than those far apart (Legendre, 1993). Therefore, we had to consider the spatial arrangements of sampling sites when testing for the relationship between traits and environment, or otherwise we could not tease apart if traits were responding to environmental variation or simply reflect spatial distribution of species. Spatial autocorrelation poses a problem for statistical analyses if sampling units are not independent (pseudoreplicates). But the method we use here (see Section 2.5.2) takes advantage of the wealth of information SAC can provide (see Legendre, 1993) to understand how climate can change organ coloration depending on where species occur (Pavoine et al., 2011).

The entries of the trait matrix were the coloration category for each individual (rows) in each organ (columns). To minimize multicollinearity, we calculated VIF (Zuur et al., 2010) and a pair‐wise correlation matrix of all organs and structures. Cardiac blood vessels, renal veins, and lumbar nerve plexus had a high VIF (>10) and a high correlation with the organs associated with them. Therefore, we excluded them from further analysis. We then tested for phylogenetic autocorrelation (or phylogenetic “signal”) in the coloration of each organ by decomposing the diversity of organ coloration categories, calculated as Rao's quadratic entropy, along the nodes of the phylogeny (Pavoine et al., 2010). This method uses tree topology to decompose trait diversity. Afterward, we tested whether the diversity of coloration categories was skewed toward the root of the phylogeny, or concentrated in a single or a few nodes (Pavoine et al., 2010). In this context, a phylogenetic signal occurs when trait diversity is skewed toward the root of the phylogeny, implying that all its descending lineages would have similar values for that trait. Similar to the spatial effect, by explicitly considering the phylogenetic structure of organ coloration, we can gain insights into not only how it evolved, but also how the effect of climatic variables on this trait varies among lineages.

#### 2.5.2 Matching organ coloration to climatic gradients

To test which set of climatic variables had the highest covariation with the coloration on the surface of internal organs of individuals, we used the RLQ ordination (Fig. S1; Dolédec et al., 1996; Dray et al., 2014). The RLQ (R‐mode Linked to Q‐mode) is a three‐table ordination technique that extends the co‐inertia approach (Dolédec & Chessel, 1994) to deal with three tables instead of two (Dolédec et al., 1996). As such, it maximizes the covariation between linear combinations of the columns of the environmental (sites by variables, **Q** matrix) and trait (species by trait, **R** matrix) matrices using a species composition matrix (species by site) as a link (**L** matrix). The first step in the RLQ is to analyze each table (**R**,** L**, and **Q**) separately using an appropriate ordination technique to deal with each type of data (Dray et al., 2014). For example, a correspondence analysis (CA) is usually applied to the **L** matrix, while a principal component analysis (PCA) is applied to the **R** matrix if it contains only continuous variables, and a principal coordinate analysis (PCoA) is used onto the **Q** matrix if it contains categorical, fuzzy, or combinations of data (Legendre & Legendre, 2012). Then, the RLQ combines these separate analyses to identify the main relationships between environmental gradients and traits mediated by species abundance or incidence (Dray et al., 2014). The result of such analysis is in terms of ordination axes that summarize the relationship between traits and environmental variables (Dolédec et al., 1996; Dray et al., 2014). The RLQ is a good method to analyze how traits relate to environmental gradients at the species level (Kleyer et al., 2012).

Here, we used an extended version of the RLQ (Fig. S1; Pavoine et al., 2011) that takes into account the spatial autocorrelation in environmental variables and phylogenetic autocorrelation in traits. This new method combines the original environmental (**E**), trait (**T**), phylogenetic distance (**P**), and geographical coordinates (**S**) matrices using factorial (=unconstrained ordination) analysis. Then, each matrix is standardized by dividing it by the square root of its first eigenvector ($\sqrt{\lambda_{1}}$). Afterward, the standardized spatial ($X_{S}^{*}$) and environmental ($X_{E}^{*}$) matrices are juxtaposed as [$X_{E}^{*}$|$X_{S}^{*}$] to become matrix **R**, while matrix **Q** is defined as [$X_{T}^{*}$|$X_{P}^{*}$] by the juxtaposition of the standardized trait ($X_{T}^{*}$) and phylogenetic ($X_{P}^{*}$) matrices (Pavoine et al., 2011). Finally, these new **R** and **Q** matrices are analyzed using the regular RLQ method (Dolédec et al., 1996).

To implement the extended RLQ, we began by first analyzing the species composition matrix (**L** matrix), which contains the presence of each individual analyzed in rows and localities as columns. This matrix was analyzed using a CA, because it can deal with both species incidence and abundance (Legendre & Legendre, 2012). Then, we proceeded to analyze the other matrices using ordination techniques. We calculated a cophenetic distance matrix (**P** matrix) from the phylogeny and calculated a weighted PCoA, derived from species weights of the CA. This analysis produced the phylogenetic eigenvectors. The reduced matrix of environmental variables (**E** matrix) was summarized using a weighted PCA, with species weights derived from CA, as it only contained continuous variables. To model space (**S** matrix), we built a neighbor matrix linking sites separated up to 318.88 km (based on the truncation distance of a minimum spanning tree). Then, we computed a PCA onto this neighbor matrix to use as spatial variables.

We calculated a distance matrix for the categories of organ coloration (**T** matrix) based on the modified Gower similarity coefficient (Pavoine, Vallet, Dufour, Gachet, & Daniel, 2009), treating ranked data as quantitative variables. Posteriorly, we tested for a relationship between environmental variables and the coloration of each organ using a multivariate version of the fourth‐corner analysis (based on the sum of all eigenvalues), to select traits with significant correlations with climatic variables. Significance was assessed using the null model 4 (Dray & Legendre, 2008; Pavoine et al., 2011). All organs, except the pericardium had significant relationship with environmental variables. Thus, we excluded this organ from further analysis. The trait distance matrix excluding the pericardium was then analyzed with a weighted PCoA, using the species weights derived from the CA.

The results of the RLQ are interpreted in terms of the first ordination axis, using Spearman rank correlation of scores of ordinal traits (levels of coloration) and Pearson correlation for the scores of continuous climatic variables along the first RLQ axis. Analyses were implemented in R v. 3.3.2 (R Core Team 2016) package ade4 (Dray & Dufour, 2007) and custom functions provided by Pavoine et al. (2011).

## 3 RESULTS

### 3.1 Phylogenetic pattern of organ coloration

Coloration on the surface of organs varied consistently among and within species (Figure 2). The coloration showed a phylogenetic pattern, but also varied throughout space within the same species. The coloration considering all organs was significantly biased toward the root of the phylogeny when we considered all organs together, but not each one separately, indicating phylogenetic signal (Table S1, Fig. S2). The greatest variation in organ coloration was found in Leptodactylidae (Figure 3), mainly because Leiuperinae have a consistent pattern of coloration on the testes. The second node with highest diversity includes all species except Brachycephaloidea (*Ischnocnema*,* Holoaden,* and *Haddadus*) and Microhylidae (*Elachistocleis*). The highest level of intraspecific variation occurred in *D. minutus* and to a lesser extent in Dendropsophryni (*Dendropsophus* and *Scinax*) as a whole (Figure 3). The organs that had the greatest diversity of coloration were the testis, heart, rectum, mesentery, peritoneum, and lung (Fig. S2).

### 3.2 Relationships between organ coloration and climatic variables

**Figure 4:**
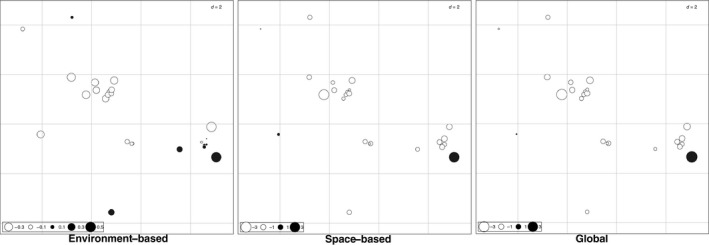
Results of the RLQ analysis visualized in space. Circles show the localities sampled. Sites in black are positively correlated, while blank sites were negatively correlated to the first RLQ axis. Size of circles indicates the absolute value of the coordinates respective to the first RLQ axis

**Figure 5:**
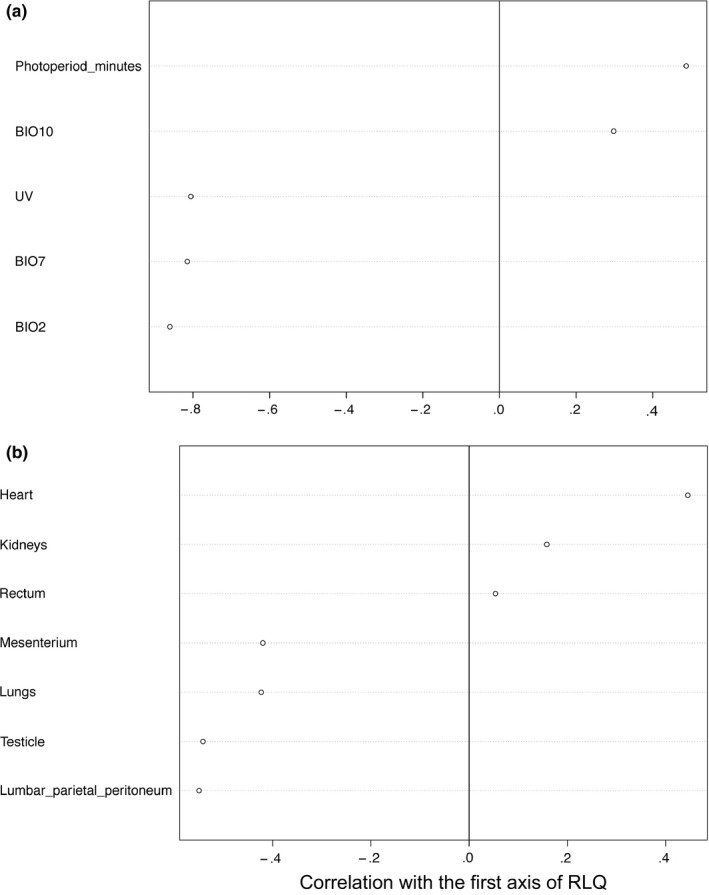
Correlations of environmental variables and organs with the first axis of RLQ. (a) Pearson correlation between the environmental variables and the coordinates of sites in the first axis. (b) Spearman rank correlations between organ coloration category (ordinal) and the coordinates of species on the first axis. Species with high coloration on the heart, kidney, and rectum occur in sites with high BIO10 (mean temperature of the warmest quarter) and photoperiod, whereas those with high coloration on the mesenterium, lungs, testicle, and peritoneum occurred in sites with high BIO2 (diurnal temperature range), BIO7 (annual temperature range), and UV‐B

**Figure 6:**
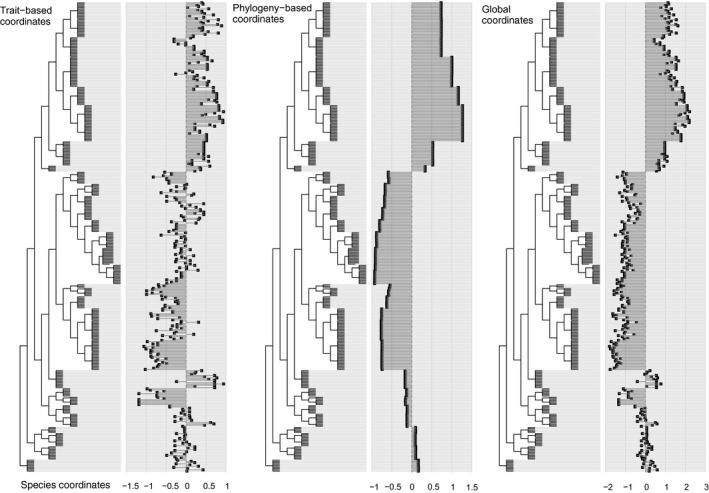
Results of the RLQ analysis (first axis) visualized on the phylogeny. The trait‐based plot represents the scores of species according to their traits plotted on the first axis of the RLQ. Phylogeny‐based plot represents the coordinates of species according to their phylogenetic relationship. The coordinates of the species in the global plot are the sum of combination of trait and phylogenetic variables

The co‐inertia of first axis of the RLQ was 0.67, which means it summarizes 67% of the total variation of both space and environment, and both traits and phylogeny. The positive side of the first axis corresponds to areas in the eastern São Paulo state, Paraná, and Goiás (Figure 4) that have high both photoperiod and mean temperature in the warmest quarter (Figure 5a). The species found in those localities have high coloration on the heart, kidneys, and rectum (Figure 5b). These species are the majority of hylids, *Rhinella schneideri*, some *Leptodactylus*, and *Proceratophrys* (Figure 6).

The negative side of the axis 1 represents localities in southern Minas Gerais and in the northwest of São Paulo state (Figure 4) that have high UV‐B, mean diurnal (BIO2) and annual (BIO7) temperature range (Figure 5a). The species found in these localities have high coloration on the testicle, lumbar parietal peritoneum, lungs, and mesenterium (Figure 5b). The species are Leiuperinae, Hylodidae, *Adenomera*, most *Leptodactylus*, and to a lesser extent the Brachycephaloidea and *Elachistocleis* (Figure 6).

## 4 DISCUSSION

Contrarily to our initial hypothesis, climatic variables influenced the coloration on each organ differently. Additionally, the internal coloration in anurans showed a non‐stationary phylogenetic component, with a stronger effect in the testicle and peritoneum. Interestingly, we also found a large intraspecific variation in coloration intensity in some lineages, mainly the Dendropsophryni tribe (*Dendropsophus* and *Scinax*).

Both diurnal and annual thermal ranges had a high covariation with the coloration on the testicle, lumbar parietal peritoneum, lungs, and mesenterium. Melanin‐containing cells protect tissues against temperature variation (Cesarini, 1996). Melanin decreases in hibernating species during the winter, due to both decreased synthesis and cellular apoptosis (Barni et al., 2002). Similarly, temperature variation reduced the hepatic pigmentation in a Neotropical anuran species (Santos et al., 2014), demonstrating that temperature is a key environmental factor controlling the amount of internal melanin. The liver has a different pigment cell type (melanomacrophages) than other organs (Franco‐Belussi et al., 2013). However, the thermal protection properties of these both cell types are provided by melanin and are thus equivalent (Franco‐Belussi et al., 2013). Melanin can also dissipate heat and is probably involved in thermoregulation in ectotherms (Cesarini, 1996). High temperature variation can disrupt the gametogenesis of anurans (Rastogi, Iela, Saxena, & Chieffi, 1976). Thus, the amount of melanin may be a trait with possible adaptive functions that help anuran species to cope with wide variation in temperature in order to protect temperature‐sensitive organs.

We found that UV‐B radiation influences coloration in the same organs as temperature range. A previous study found that short‐term exposure to low doses of UV‐B increases internal coloration by increasing melanin production and dispersion (Franco‐Belussi et al., 2016). UV radiation can have genotoxic effects in cells by disrupting DNA (Ortonne, 2002). Melanin provides protection against solar radiation, by dissipating solar energy in the form of heat (Ortonne, 2002). As a result, tyrosinase activity increases in melanin‐containing cells, which increases melanin production to protect cells against UV radiation (Friedmann & Gilchrest, 1987). Therefore, species that occur in sites with high UV‐B incidence potentially have greater production of melanin as a mechanism to deal with its deleterious effects.

UV radiation can have deleterious effects on anurans (Lipinski et al., 2016). Consequently, species that occur in places with high UV incidence could have developed more melanin on the testicles as a way to protect their germinal epithelium (Franco‐Belussi et al., 2016), as damage in the gametes can influence the reproductive fitness of individuals. For example, hylodids had a large amount of melanin on the testicles and are restricted to the Atlantic rainforest. This region has the same degree of UV incidence of the northwest of São Paulo, where swamp frogs of the subfamily Leiuperinae occur. As a consequence, these species developed similar strategies to deal with elevated UV‐B variation by having high amount of coloration on the testicles. Also, having a high amount of melanin on the testicles may allow species to be active during the day, such as dendrobatids (Grant et al., 2006), hylodids, or at dusk, like *Pseudopaludicola* and some *Physalaemus* (Vasconcelos & Rossa‐Feres, 2005). Conversely, species lacking melanin on the testicles are mainly active at night (e.g., Hylidae and Leptodactylidae; Vasconcelos & Rossa‐Feres, 2005).

The coloration on the heart, kidneys, and rectum had a high covariation with photoperiod. Additionally, the intensity of coloration on the heart and kidneys of hylids tended to be lower than in the testicles of Leiuperinae, showing a phylogenetic signal. The effects of photoperiod on internal coloration are poorly known. However, photoperiod directly influences the endocrine system (Breet, 1979), which can indirectly alter melanin amount. An increase in photoperiod (e.g., 18:6 vs. 9:15 light:dark) promoted whitening of fish's skin by increasing the secretion of MCH (Lyon & Baker, 1993; Guinés et al., 2004). The adaptive value of coloration on the heart, kidneys, and rectum is still not clearly understood (see Colombo, Berlim, Delmas, & Larue, 2011), but it is probably related to the functions of the melanin molecule, which mainly acts as antibiotic, light absorption (e.g., photoprotection), cation chelator, and free radical sink (Riley, 1997). Consequently, photoperiod may indirectly affect internal melanin coloration of ectotherms by its organismal‐wide effect on hormones, differently from temperature and UV‐B, which directly interfere on melanin at the cellular level.

We found that the amount of melanin on a given organ covaried not only with environmental variables, but also with organ physiology and species phylogenetic relationships (see also Provete et al., 2012). Unexpectedly, the coloration on the surface of each internal organ responded differently to climatic variables and depending on the phylogenetic lineage of species. Melanocytes have distinct physiology depending on the external coloration of the animal. For example, pigmented cells on the peritoneum of fish respond to hormones, such as melatonin and epinephrine, by either aggregating or dispersing depending on their cutaneous coloration (Sköld, Svensson, & Zejlon, 2010). Although the pigmented cell type is the same in these organs, the response of melanin to the various climatic factors may vary according to organ physiology. These results reinforce the role of physiological responses on pigmented cells. However, the response of pigmented cells in different organs may be through distinct mechanisms, such as aggregation or dispersal of melanin granules. Our results documented an interesting pattern of variation of internal organ coloration, which may guide future experimental studies to test how specific physiological mechanisms regulate the response of melanocytes on several organs to climatic variables.

To conclude, our findings can help interpret the results of experimental studies testing the effect of UV, temperature, and photoperiod on internal melanin pigmentation in ectotherms under a new perspective. They may also reconcile apparently conflicting experimental results about the effect of temperature on internal pigmentation (see Corsaro, Scalia, Sinatra, & Sichel, 1990; Santos et al., 2014). For example, Corsaro et al. found that hypothermy increased melanin pigmentation in *Pelophylax lessonae* during the winter (10°C) in relation to the summer (20–25°C) in southern Italy. Conversely, Santos et al. found that both low (18.9°C) and high (35.1°C) temperatures decreased melanin pigmentation in *Physalaemus nattereri* in the northwestern São Paulo state, Brazil. These apparently contrasting results can be explained by the different phylogenetic lineages to which these species belong and the higher annual thermal range in the Tropics. Our results suggest that the effect of climatic variables on melanin varies not only among internal organs, but also among phylogenetic lineages and regions across space, which might explain the different results of experiments conducted in the temperate vs. tropical regions.

## ACKNOWLEDGMENTS

The study was supported by a research grant from the São Paulo Research Foundation (FAPESP) (#2015/12006‐9 and 05/02919‐5) to C.O. and postdoc fellowships to LFB (#2014/00946‐4) and DBP (#2016/13949‐7). LFB was also supported by a CAPES‐PNPD postdoc fellowship during the final preparation of this manuscript. T. Gonçalves‐Souza and F. R. da Silva helped interpreting the analysis output. L. R. S. Santos, I. A. Martins, S. A. César, R. M. Moresco, and R. Zieri helped with specimen sampling. L. R. S. Santos provided additional specimens. B. Vilela and D. P. Silva kindly prepared the map.

## DATA ACCESSIBILITY

All data and R code used to run the analyses are deposited in FigShare (Franco‐Belussi et al., 2017) at https://doi.org/10.6084/m9.figshare.4707187.v1.

## CONFLICT OF INTEREST

None declared.

## AUTHORS’ CONTRIBUTIONS

LFB collected the data. DBP analyzed and curated the data and prepared the figures and supplementary material. LFB and DBP prepared the first draft of the manuscript. CO designed the study and contributed chemicals and equipment. All authors read and approved the final manuscript.
