# Supplementary material for: Environmental correlates of internal coloration in frogs vary throughout space and lineages
Source: Ecol Evol. 2017 Oct 3;7(22):9222–33. doi: 10.1002/ece3.3438 (PMC5696405; doi:10.1002/ece3.3438)
Supplement: Supplementary file 1 [file ECE3-7-9222-s001.pdf]

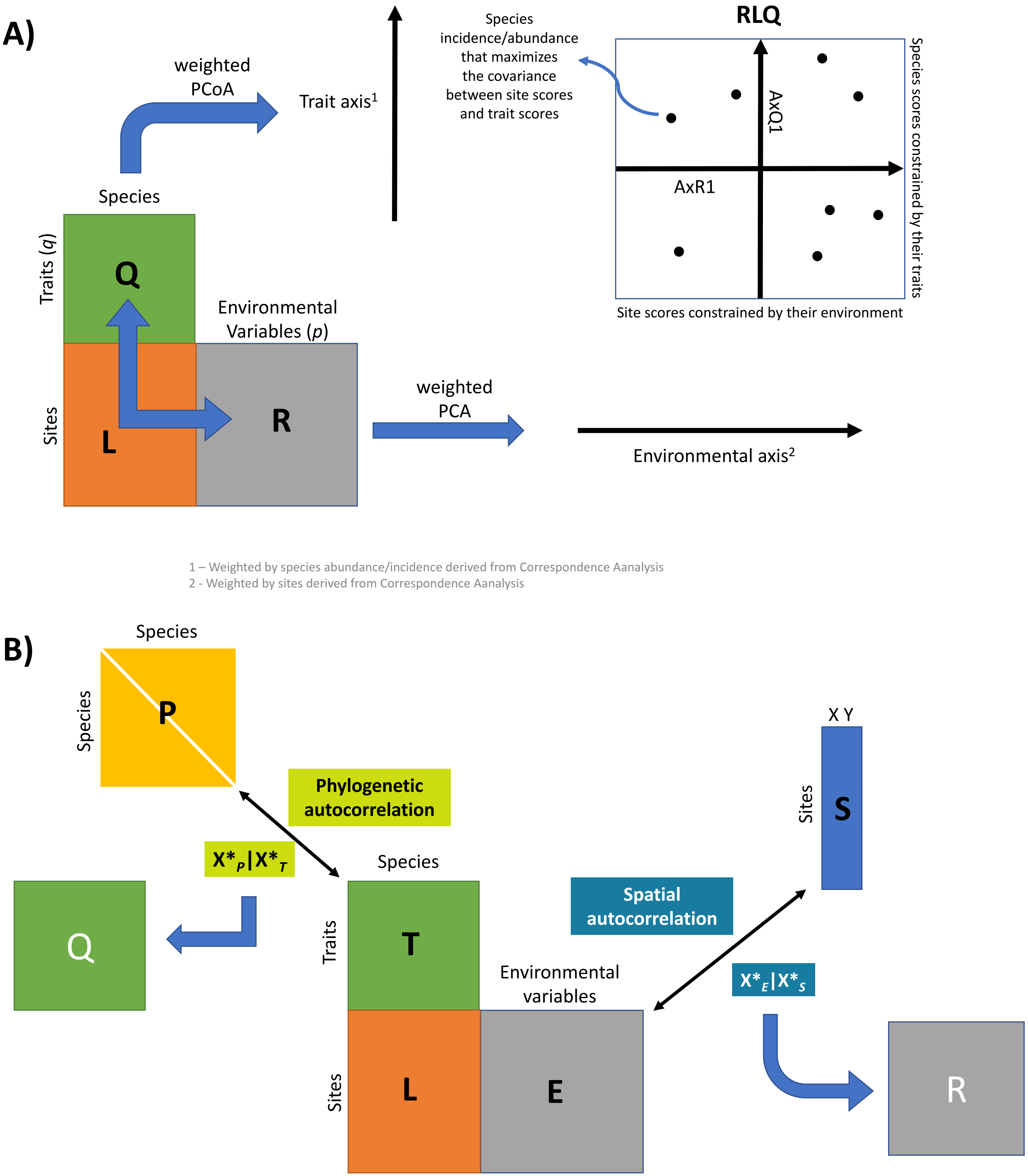
A)
weighted
PCoA
Trait axis¹
Species
incidence/abundance
that maximizes
the covariance
between site scores
and trait scores
RLQ
AxQ1
AxR1
Species scores constrained by their traits
Site scores constrained by their environment
Species
Traits (q)
Q
Environmental
Variables (p)
Sites
L
R
weighted
PCA
Environmental axis²
1 – Weighted by species abundance/incidence derived from Correspondence Aanalysis
2 - Weighted by sites derived from Correspondence Aanalysis
B)
Species
Species
P
Phylogenetic
autocorrelation
X*P|X*T
Q
Species
Traits
T
X Y
Sites
S
Spatial
autocorrelation
Environmental
variables
X*E|X*S
Sites
L
E
R
