# Supplementary figures and images for: Environmental correlates of internal coloration in frogs vary throughout space and lineages
Source: Ecol Evol. 2017 Oct 3;7(22):9222–33. doi: 10.1002/ece3.3438 (PMC5696405; doi:10.1002/ece3.3438)

**A**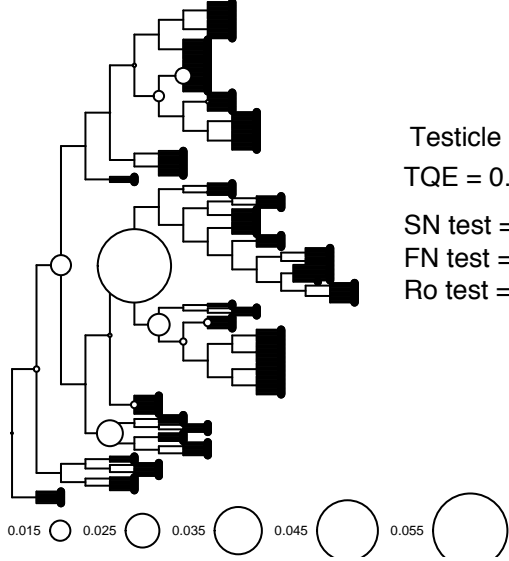**B**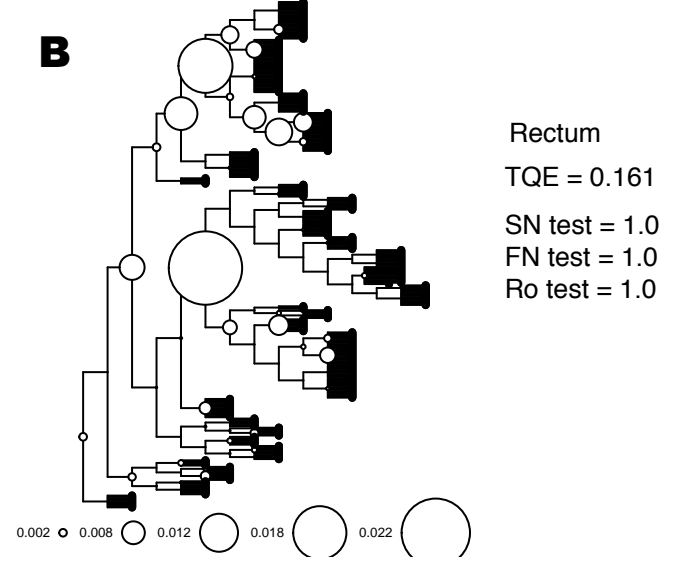**C**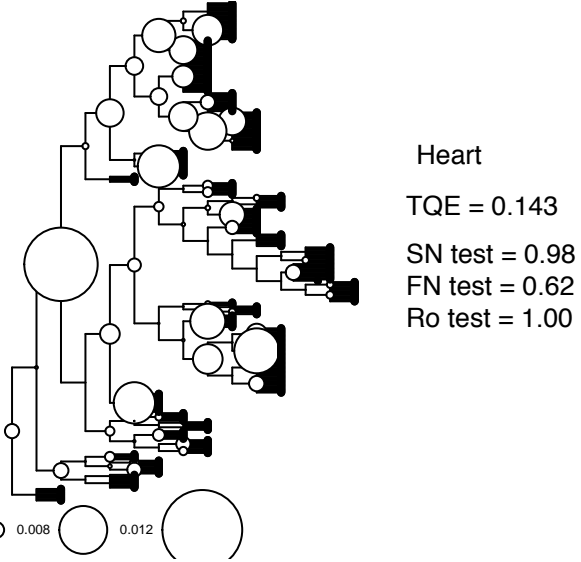**D**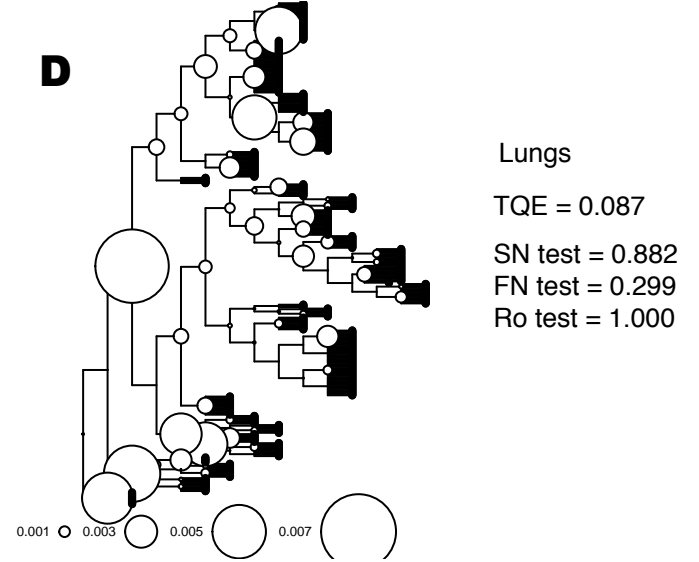**E**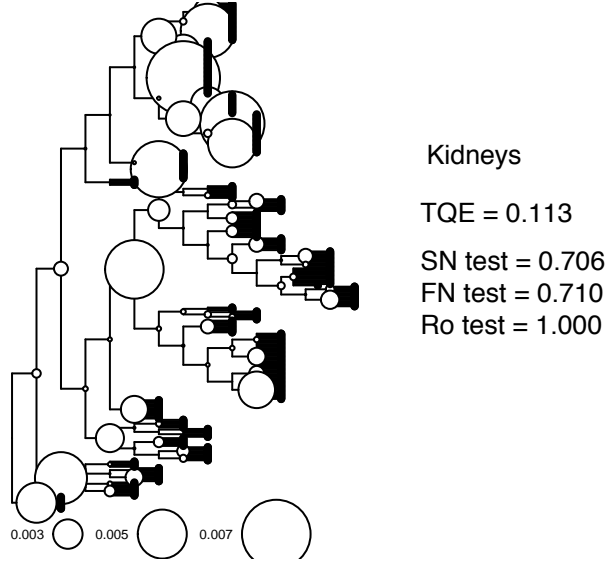**F**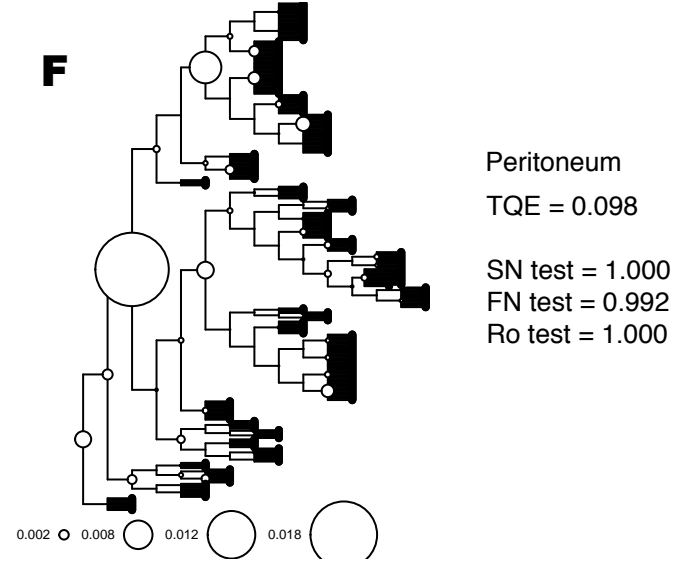**G**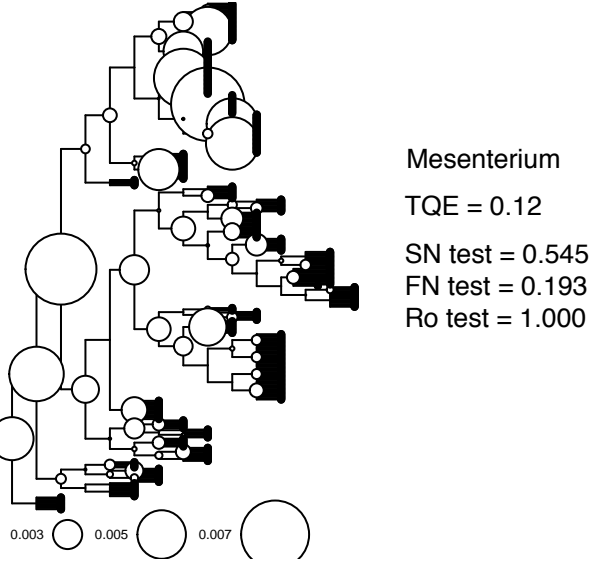

Supplement: Supplementary file 2 [file ECE3-7-9222-s002.pdf]
