# Supplementary material for: Environmental correlates of internal coloration in frogs vary throughout space and lineages
Source: Ecol Evol. 2017 Oct 3;7(22):9222–33. doi: 10.1002/ece3.3438 (PMC5696405; doi:10.1002/ece3.3438)
Supplement: Supplementary file 3 [file ECE3-7-9222-s003.docx]

|  | Deviation  from theoretical value | *P*-value | Conclusion |
| --- | --- | --- | --- |
| Testicle | 23.84 | 1 | NS |
| Heart | 12.95 | 1 | NS |
| Lungs | 8.09 | 1 | NS |
| Kidney | 9.63 | 1 | NS |
| Rectum | 21.02 | 1 | NS |
| Mesenterium | 12.62 | 1 | NS |
| Peritoneum | 19.25 | 1 | NS |
| All organs | 38.65 | <0.001 | S |
